# Do Age and Sex Play a Role in the Intraocular Pressure Changes after Acrobatic Gymnastics?

**DOI:** 10.3390/jcm10204700

**Published:** 2021-10-13

**Authors:** Javier Gene-Morales, Andrés Gené-Sampedro, Alba Martín-Portugués, Inmaculada Bueno-Gimeno

**Affiliations:** 1 Research Group Prevention and Health in Exercise and Sport (PHES), University of Valencia, St. Gascó Oliag 3, 46010 Valencia, Spain; javier.gene@uv.es; 2 Research Institute on Traffic and Road Safety (INTRAS), University of Valencia, St. Serpis 29, 46022 Valencia, Spain; 3 Department of Optics, Optometry & Vision Science, University of Valencia, St. Dr Moliner 50, 46100 Burjassot, Spain; alba.mv17@hotmail.com

**Keywords:** physical exercise, sport, acrobatic gymnastics, baseline intraocular pressure, central corneal thickness, ocular health, tumbling skills, hand balance

## Abstract

To evaluate the effects of an acrobatic gymnastics (AG) training session on intraocular pressure (IOP), a familiarization session was employed to confirm the participant’s suitability for the study. Forty-nine gymnasts (63.27% females, 18–40 years old) voluntarily agreed to participate. As age, sex, baseline IOP, and central corneal thickness (CCT) were considered as potential predictors of the IOP variations, in the second session measurements of the above parameters were taken before and after 90 min of AG. A mixed-factorial analysis of variance evaluated differences. Linear regression was conducted to potentially predict the IOP variation with the exercise. After the scheduled exercise, highly significant (*p* < 0.001, effect size: 0.73) reductions in IOP, but no significant changes in CCT (*p* = 0.229), were observed. IOP was significantly modified in males, older than 25 years, and subjects with baseline IOP > 14 mmHg (*p* ≤ 0.001, effect sizes: 0.57–1.02). In contrast, the IOP of females, younger participants, and subjects with baseline IOP ≤ 14 mmHg was not significantly modified (*p* = 0.114). With the regression analyses, we concluded that both sex and baseline IOP levels were significant predictors of the IOP fluctuation with AG. These findings could be of interest for gymnasts, coaches, ophthalmologists, and/or optometrists in the prevention and control of risk factors associated with glaucoma.

## 1. Introduction

Intraocular pressure (IOP) and its fluctuations are still recognized as the main modifiable factor in the control, management, and prevention of glaucoma [1,2,3]. IOP can fluctuate due to different internal and external factors. Among them, age and sex are acknowledged factors that condition IOP [4,5]. Additionally, corneal thickness [6] and baseline IOP levels [2,7] have been identified to play a role in the short-term IOP fluctuations. As far as we know, no previous research has analyzed the potential effects of baseline IOP levels and corneal thickness (CCT) on the IOP fluctuations caused by acrobatic gymnastics (AG) exercise.

Exercise is a key external factor that modifies intraocular pressure [3,4,5,8,9] and cardiovascular parameters [10]. More specifically, aerobic, continuous exercise such as running or cycling at low to moderate intensities has proven to acutely reduce IOP [8,11,12,13]. Regarding resistance exercises involving muscular strength such as weightlifting, controversial results appear in the scientific literature, with many studies ensuring IOP elevations [14,15,16,17,18,19,20] and other studies reporting IOP reductions due to the exercise effect [21,22,23,24,25,26,27,28,29,30]. As shown in previous expert literature, recovery of pre-exercise IOP values could take from several minutes after resistance exercises to up to one hour after aerobic exercise [4,8]. In addition to the exercise methodology itself, certain positions during the activity such as head-down positions could increase IOP [3,5,8]. Considering the above concerns, it remains necessary to study sport disciplines that in their practice combine the aerobic and muscular systems and changes of position, such as AG. Nevertheless, knowledge on AG remains incomplete [31], especially in terms of ocular adaptations. No previous studies dealing with IOP variations after AG were found.

AG is growing in popularity among different age groups [31,32]. AG is a combined activity that can be performed in pairs or groups and includes static elements such as balances and figure holds (hand balances, bridges, splits, human pyramids) and dynamic elements such as partner lifts, throws with complex somersaults and twists, and tumbling skills [33,34,35]. This motor and social sport requires high levels of strength, flexibility, balance, agility, coordination, speed, and cardiovascular performance [36]. Due to the aforementioned topics, it is scientifically necessary to evaluate the IOP acute adaptations that could occur after an AG session, to obtain a better understanding of the effects of this activity that could not be reached within a laboratory environment. Furthermore, the question arises as to whether sociodemographic and ocular variables such as sex, age, baseline IOP, and baseline CCT could play a role in the IOP variations.

The main aim was to evaluate IOP and CCT variations after an AG session. Additionally, a set of demographics (age and sex) and ocular parameters (baseline IOP and CCT) were considered as potential predictors of the IOP variation due to the exercise effect (difference between post-exercise and pre-exercise intraocular pressure values).

We hypothesized that exercise would reduce the IOP and CCT would remain unchanged. We also expected to find that the independent variables (age, sex, baseline IOP, and CCT) would affect the IOP variations.

## 2. Materials and Methods

An observational, prospective, longitudinal study was conducted to compare the IOP and CCT of gymnasts pre- and post-exercise. Additionally, the prediction potential of age, sex, baseline IOP, and CCT on the variation of IOP was addressed. We conducted the study in conformity with the Code of Ethics of the World Medical Association (Declaration of Helsinki [37]), and ethical approval was provided by the Research Ethics Committee on human research of the University of Valencia (H1499867368458). The study was also approved by the Club Dynamic Gym of Manises (Valencia, Spain). The subjects were informed of the study characteristics and protocols, and signed, informed consent was obtained from all the participants at the beginning of the procedures. Participants were free to withdraw from the study at any time. Data were confidential and participation was anonymous, implying no potential risks for the integrity of the subjects apart from those derived from the physical activity.

### 2.1. Participants

The sample size was determined by a priori power analyses, assuming an α of 0.05, power levels (1-ß) of between 0.80 and 0.95, a non-sphericity correction of ε = 1, and an effect size of f(V) = 0.45 for ANOVA tests and f^2^ = 0.24 for the regression analyses. Thus, 49 participants were recruited for this study. Main inclusion criteria were: (1) older than 18 and younger than 40 years old, (2) experience with acrobatic gymnastics of at least 6 months and performing at least 2 days per week, (3) no musculoskeletal issues, (4) baseline IOP between 10.00 and 21.00 mmHg, (5) normal anterior chamber depth, (6) no history of ophthalmic laser procedures, ocular surgery, traumatism, or use of topic/systemic medications potentially affecting the IOP. Subjects with a family history of glaucoma and/or contact lens wearers were excluded from this study.

At the beginning of the study, 51 athletes were recruited, but only 49 met the criteria (18 male and 31 female). All these subjects voluntarily agreed to participate in the study. Participants were classified into two groups according to their age: (1) adults (>25 years old) and (2) young adults (≤25 years old) [38]. Additionally, three more groups were formed regarding the baseline IOP levels (low, medium, and high). For such purpose, baseline IOP was divided into terciles (with limits at 14 and 17 mmHg) as previously reported [2,7]. Further characteristics of the sample, including demographics and spherocylindrical refraction values, are reflected in Table 1. The spherocylindrical refraction values were converted to power vector notation (M, J0, and J45). Refractive error was determined in terms of (1) the spherical equivalent (M component) and (2) a pair of Jackson Crossed Cylinder lenses oriented at 0°/90° (J0 component) and 45°/135° (J45 component) for determination of astigmatism. Refractive error was measured to characterize the sample considering its potential influences on IOP [39].

All participants were instructed to avoid alcohol/tobacco consumption and to not perform vigorous exercise 24 h before any programmed session. They were asked to sleep for at least 8 h, to not consume stimulants, to not drink more than 1 L of liquids [4], and to not perform prolonged near-viewing activities within the 3 h before the trials [40].

### 2.2. Procedures

All procedures were conducted in the same gymnastic facilities by the same researchers (one optometrist (in charge of the measurements) and one sports scientist (responsible for the gymnastics session)). All data were collected in a thermoneutral environment (~22 °C and ~60% humidity), under the same lighting, and at the same period (between 7:20 p.m. and 9:10 p.m.) to reduce the effects of circadian rhythm variations in the eyes [41]. Measurement tools were installed in a room next to the training facilities to improve access and performance of techniques. Two sessions separated by 1 week were scheduled: one for assessment of sociodemographic data, participants’ characteristics, and systematized ophthalmological examination at baseline, and a second session to carry out all experimental procedures to evaluate the dependent variables before and after the AG session.

In the first session, an ocular examination was performed to ensure the validity of participants, including measurements of best-corrected visual acuity, subjective refraction, IOP (Auto Kerato-Refracto-Tonometer TRK-2P; Topcon^®^, Tokyo, Japan), stereopsis, motility, and biomicroscopic anterior eye segment examination (Slit Lamp SL-D4, Topcon Europe Medical BV, The Netherlands). Objective refraction was measured with the Auto Kerato-Refracto Tonometer (TRK-2P, Topcon^®^, Tokyo, Japan) and was followed by a subjective refinement.

In the second session, pre-exercise eye parameters were measured 5 to 10 min before starting the exercise. All subjects underwent the same 90-min acrobatic gymnastics training session (as reflected in the previous section, for further information on the specific characteristics of this type of sport). IOP and CCT were measured again 5 to 10 min after finishing the exercise.

#### Intraocular Pressure and Central Corneal Thickness

As above reflected, IOP and CCT were measured in mmHg and microns, respectively, with the Auto Kerato-Refracto-Tonometer TRK-2P (Topcon^®^, Tokyo, Japan). This non-contact instrument is composed of Rotary Prism Technology and provides unmatched accuracy and reliability as well as permitting accurate and reliable measurements with a pupil as small as 2 mm in diameter. The device uses optical pachymetry to determine CCT, which involves using a tangential slit of light directed onto the cornea at a known angle. The illuminated slit is measured, and corneal thickness is calculated using trigonometry. All parameters, including horizontal and vertical alignment and vertex distance, were determined by the instrument. Additionally, TRK-2P allows adjusting the value of pneumotonometry with pachymetry, so that it automatically adjusts the IOP value based on corneal thickness [42]. The measurements were taken using the full screening mode, which includes intraocular pressure, keratometry, autorefraction, and pachymetry values. Three readings for each patient were obtained, averaged, and recorded.

Measurements were taken in both eyes in this study. Right eye measurements were used since no significant difference (*p* = 0.112) was observed between the eyes.

### 2.3. Statistical Analysis

First, a basic data curation was performed, and descriptive statistics of the sample features were calculated. Variation of IOP was calculated as post-exercise IOP minus pre-exercise IOP, which, in turn, was converted to a percentage (Δ%). Normality of data distribution and homoscedasticity was assessed through the Shapiro–Wilk and Levene tests, respectively. Data showed a normal-Gaussian distribution with homogeneous variances.

At this point, a mixed factorial analysis of variance (ANOVA), with the exercise (baseline and post-exercise measurements) as the within-subject factor, and sex (male, female), age (young adult, adult), and baseline IOP levels (low, medium, high) as the between-subject factors, was used to evaluate the effects of the exercise as well as to assess differences in the study-dependent variables. Effect size was evaluated with eta partial squared (ƞp²), where 0.01 < ƞp² < 0.06 constitutes a small effect, 0.06 ≤ ƞp² ≤ 0.14 constitutes a medium effect, and ƞp² > 0.14 constitutes a large effect. Pairwise post hoc comparisons were evaluated using Bonferroni correction. The effect size for post hoc comparisons was calculated as Cohen’s *d* with Hedges’ corrections to avoid biases due to sample size or standard deviation differences [43]. This corrected value is reported as unbiased Cohen’s *d* (*d*_unb_) [44], with *d*_unb_ < 0.50 constituting a small effect, 0.50 ≤ *d*_unb_ ≤ 0.79 a moderate effect, and *d*_unb_ ≥ 0.80 a large effect [45].

Afterward, Multiple Linear Regression analyses (MLR–method: enter) were carried out for the variation of intraocular pressure (difference between post-exercise and pre-exercise IOP values). Two models’ fit were tested as potential predictors of the IOP variation, one including socio-demographic (age and sex) and one including ocular variables (baseline levels of IOP and CCT).

All the statistical analyses were carried out using the software IBM SPSS Statistics for Macintosh (Version 26.0; IBM Corp., Armonk, NY, USA), while statistical power analyses were carried out with the software G*Power (Version 3.1.9.6; [46]). The level of statistical significance was set at *p* < 0.05, and tendencies were identified from 0.05 ≤ *p* ≤ 0.13.

## 3. Results

The ANOVA performed on IOP revealed a significant effect of the exercise (F[1, 43] = 33.77, *p* < 0.001, ƞp² = 0.46), the interaction exercise*sex (F[1, 43] = 6.53, *p* = 0.015, ƞp² = 0.14), and exercise*age (F[1, 43] = 7.76, *p* = 0.008, ƞp² = 0.17). The interaction exercise*baseline IOP levels resulted non-significant, although with medium effect size (F[2, 43] = 1.70, *p* = 0.196, ƞp² = 0.08). All the rest of the interactions analyzed were not significant (*p* > 0.05). Regarding the CCT, the ANOVA revealed a non-significant effect of exercise (F[1, 43] = 3.97, *p* = 0.05, ƞp² = 0.09), or for any of the interactions analyzed (exercise*sex: F[1, 43] = 3.62, *p* = 0.064, ƞp² = 0.09; exercise*age: F[1, 43] = 0.70, *p* = 0.407, ƞp² = 0.02; exercise*baseline IOP levels (F[2, 43] = 0.24, *p* = 0.788, ƞp² = 0.01). Table 2 presents the general results of the sample. It is worth highlighting that, while IOP was significantly modified (*p* < 0.001), as a consequence of the exercise, with a moderate-large effect size (*d*_unb_ = 0.73), CCT showed non-significant differences from pre- to post-exercise experimental points (*p* = 0.229).

### 3.1. Between-Group Comparisons

Bonferroni’s post hoc comparisons for the IOP and CCT results are presented in Table 3 (sex), Table 4 (age), and Table 5 (baseline IOP levels grouping). First, regarding between-sexes comparisons, significant differences were found in pre- (*p* = 0.01, *d*_unb_ = 0.59) and post-exercise intraocular pressure (*p* = 0.04, *d*_unb_ = 0.45), but not in the CCT (pre-exercise, *p =* 0.097; post-exercise, *p* = 0.071). Highly significant differences were detected between sexes in the value of the variation of IOP (Δ%), with a significantly higher reduction found in males (mean difference (m.d.) 1.60 mmHg, 95% CI [1.11–2.13], *p* < 0.001, *d*_unb_ = 1.50). Concerning the pre- and post- comparison (within-group comparison), on the one hand, male athletes obtained a significant decrease in IOP with a large effect size (*d*_unb_ = 1.02). On the other hand, the variation of this variable was non-significant in females (*p* = 0.312). It is also remarkable that CCT was significantly modified (*p* = 0.007) from pre- to post-exercise in females, with the effect size being negligible (*d*_unb_ = 0.03).

The post hoc analyses performed for age showed significant between-group differences in the post-exercise IOP values (m.d. 1.17 mmHg, 95% CI [1.12–1.22], *p* = 0.016, *d_unb_* = 0.50), but not in the pre-exercise values (m.d. 0.02 mmHg, 95% CI [0.01–0.05], *p* > 0.05). Additionally, both age groups showed a statistical tendency of significantly different IOP variation (Δ%) with moderate effect size (m.d. 0.75 mmHg, 95% CI [0.09–0.98], *p* = 0.07, *d*_unb_ = 0.50). Only the subjects over 25 years old presented significant (*p* < 0.001) IOP fluctuations from pre- to post-exercise with a moderate-large effect size (*d*_unb_ = 0.78). The young adults did not show significant fluctuations with the exercise (*p* = 0.154). No significant changes were observed for either of the groups in terms of the CCT (young adults: *p* = 0.605; adults: *p* = 0.243).

Regarding the baseline IOP, significant differences were found in the post-exercise IOP values, as shown in Table 5. In fact, IOP variation (Δ%) showed significantly lower values in the participants with lower baseline IOP (≤14.00 mmHg) than those with higher IOP at baseline (≥17.00 mmHg; m.d. 1.60 mmHg, 95% CI [0.37–2.83], *p* = 0.008, *d*_unb_ = 0.96). The IOP variation (Δ%) in subjects with medium baseline IOP and those with higher IOP did not reflect statistical differences (m.d. 0.37 mmHg, 95% CI [0.28–1.87], *p* = 0.420, *d*_unb_ = 0.18). Furthermore, while subjects with moderate (between 14.01 and 16.99 mmHg) and higher baseline IOP displayed significant (*p* ≤ 0.001) IOP decreases with moderate effect sizes (*d*_unb_ from 0.57 to 0.74), subjects with lower baseline IOP did not show statistically significant differences (*p* = 0.114) with a small effect size (*d*_unb_ = 0.25).

**Figure 1:**
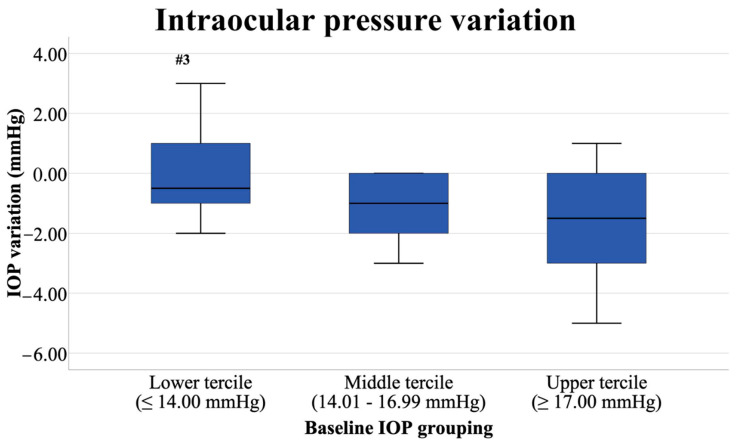
Intraocular pressure variation (post-exercise IOP minus pre-exercise IOP) of each of the three groups formed considering the baseline IOP levels (lower: *n* = 18; middle: *n* = 17; upper: *n* = 14). Values of the vertical axis (IOP variation) are presented in mmHg. The symbol “#3” highlights significant differences with the Upper-Tercile Group (*p* = 0.008, *d*_unb_ = 0.96).

Differences in IOP variation (post-exercise minus pre-exercise) of each of the three groups of participants that were subdivided by IOP values can be found in Figure 1. It is worth mentioning that some subjects of the Lower-Tercile Group (baseline IOP under 14.00 mmHg) and a few of the Upper-Tercile group (baseline IOP over 17.00 mmHg) had their IOP increased due to the exercise effect, as can see in the boxplots on the left and right. Significant differences were encountered between the Lower- and Upper-Tercile Groups (*p* = 0.008, *d*_unb_ = 0.96).

### 3.2. Regression Analyses

Multiple linear regression was calculated to potentially predict the IOP variation based on different features of the sample (age, gender, levels of baseline IOP, and levels of baseline CCT). A significant regression equation was found (F[3, 45] = 10.159, *p* < 0.001, with an adjusted R^2^ of 0.433). Baseline CCT and age were discarded from the equation due to non-significant results. The predicted variation of IOP was equal to 1.430 (sex)–0.270 (baseline IOP), where age is measured in years and the baseline IOP in mmHg. Regression analyses’ models are displayed in Table 6, where the significant model and its coefficients are described. Model 2 was retained, as it was the one with the greatest prediction potential. This model predicted 43.3% of the variance in IOP. Sex and baseline IOP levels were significant (*p* = 0.001, and *p* = 0.007, respectively) predictors of the test outcomes. As shown in Table 6, while the baseline IOP levels were negatively correlated with the IOP variation, sex showed a positive correlation.

## 4. Discussion

To the best of our knowledge, this is the first study aimed at evaluating the effect of an AG session on IOP. Additionally, a set of variables were selected to potentially predict the variation of IOP. The most notable findings were that a session of AG significantly reduced the IOP values, but did not significantly modify CCT (see Table 2), which is consistent with most previous studies on the effects of dynamic exercise on IOP [4,5,8] and confirms our first hypothesis. The small changes observed in CCT, such as those detected in females, could be due to physiological diurnal variations [47]. Additionally, it is worth highlighting that sex and baseline IOP levels were significant predictors of the fluctuation on IOP due to the exercise (see Table 6), which only partially confirms the second hypothesis. Accordingly, male gender and lower baseline IOP demonstrated in a previous study a possible association with visual field progression [48].

Bearing the aforementioned results in mind, it is worth discussing the outputs of this research under the light of other empirical evidence that addressed the influence of the independent variables selected in this study (sex, age, baseline IOP) on intraocular pressure. However, caution should be applied when comparing different methodologies of exercise and it should be borne in mind that the results presented in this study concern specifically acrobatic gymnastics.

First, sex could be a potential factor conditioning intraocular pressure due to sex hormones and genetic variants [49,50,51]. However, the findings encountered in the scientific literature are not consistent. Our results suggest that significant differences exist in both the baseline and post-exercise IOP values (see Table 3). Furthermore, while males had their IOP significantly modified due to the exercise effect, the intraocular pressure of females did not significantly change. This is in contrast with authors who encountered non-significant differences between sexes in the IOP changes due to treadmill running and isometric efforts [52,53]. On the other hand, the results presented concerning the sex of the participants are consistent with previous research that encountered differences between sexes [54,55,56,57,58] or identify sex as a confounding variable in the relationship between exercise and glaucoma [59]. More specifically, Vera et al. [60] detected further IOP fluctuations in males compared with women after isometric squats. Further research needs to be done in this regard eliminating confounding variables to elucidate if there is an actual difference in the IOP response to exercise between sexes and the origin of these differences.

Age has been widely studied as a conditioning factor of the IOP with significant positive correlations [6,52,55,61]: Only one study was found reporting non-significant correlations between age and IOP [62]. The age of 40 is recognized by the American Academy of Ophthalmology as the cutoff criterion to start comprehensive medical eye evaluation screening [63]. Due to this, only subjects under 40 years old were selected for the study. Although age was excluded from the prediction equation and was not correlated with IOP variations, significantly different behaviors were observed in the IOP of young adults under 25 years old and adults over 25. The fact of not finding a significant correlation with age in the present study could be due to the age of the sample being limited to subjects under 40 years, with studies reporting that the significant increase in baseline values occurs after the age of 40 [64]. This is interesting and coincides with the information presented in Table 4. While the baseline IOP of both groups (under 40 years old) was not significantly different, the after-exercise IOP showed significant between-group differences with a moderate effect size (*d*_unb_ = 0.50). These results suggest that once finished with the effort, the young adults under 25 years old return faster to pre-exercise values than adults over 25 years old. This could be due to the compensatory mechanisms in charge of maintaining tissue stability [2], which may function better in younger subjects, as demonstrated in rats [65].

As for the third independent variable included in this study, it is worth highlighting that IOP followed different behaviors in subjects with medium and high baseline IOP compared to subjects with lower baseline IOP (see Table 5). This is consistent with previous research that encountered larger fluctuations in subjects with higher baseline IOP and less pronounced fluctuations in subjects with lower baseline IOP [2,7,66]. More specifically, larger post-exercise decreases in subjects with higher pre-test values are reported by the expert literature [54,67,68,69]. In contrast, one study encountered a negative significant correlation between baseline IOP and its change (elevation) after an incremental running test [70] and other non-significant correlations [71]. The results presented are to be considered of relevance, bearing in mind that subjects with lower IOP are more susceptible to optical nerve damage with fluctuations [7,48]. It could be stated that the baseline level of IOP influences the post-exercise IOP and, therefore, this should be a factor to consider in the management of subjects with glaucoma risk factors.

Finally, the analysis and comparison with animal studies could shed some light on the behavior of IOP with exercise. For instance, Castro et al. [72] found positive results in the IOP of rats on a high-fructose diet with treadmill exercise at low intensity. These authors proposed as potential underlying mechanisms improved insulin sensitivity, reduced arterial pressure, and diminished peripheral sympathetic modulation [72]. Additionally, one study reported that swimming can reverse the negative impact of aging on the optic nerve function of rats [73]. As reported by previous expert literature, exercise-related IOP diminishments could be related to lower norepinephrine concentrations, increased colloid osmotic pressure, co-action of nitric oxide and endothelin after exercise, and the association with a β2-adrenergic receptor gene polymorphism [74,75,76]. Future studies should evaluate the specific mechanisms that led to lower post-exercise IOP with AG.

### Limitations and Future Directions

Although all the procedures carried out in this study were carefully designed and supervised, several limitations should be listed. Validated non-contact air-puff tonometry was chosen as it is easy to use and does not require the use of anesthesia [77,78]. However, one should bear in mind that the values presented in this study only reflect pre- and post-exercise values. In this regard, continuous monitoring devices [79] would provide the scientific literature with relevant information on what exactly happens during the practice. Additionally in this concern, future studies should address the time needed for IOP to return to pre-exercise values with similar exercise procedures. As per the results on the different IOP behaviors depending on the age of subjects, it could be interesting to include adults over 40 years in a similar study design. Finally, and as presented in the introduction, the importance of field-based studies like this is unnegotiable; however, it could be of great scientific interest to continuously monitor IOP while performing somersaults and/or tumbling skills in a controlled laboratory environment.

## 5. Conclusions

In summary, IOP significantly decreased and CCT remained unchanged from pre- to post-exercise. The IOP of males was lowered from baseline to the end of the study. On the other hand, females did not reflect IOP changes. Similarly, the IOP of adults was further reduced compared to young adults. Finally, subjects with higher IOP at baseline (middle and upper terciles) had more pronounced decreases than the participants with lower IOP. Sex and baseline intraocular pressure were obtained as significant predictors of IOP variation.

Taken together, the analyses presented in this article shed some light on the behavior of specific ocular parameters after exercise. The combination of findings presented herein could be of interest for the programming of physical exercise for gymnastics coaches and ophthalmologists or optometrists in the prevention, management, and control of risk factors associated with IOP and glaucoma.

## Figures and Tables

**Table 1:** Characteristics of the general sample (*n* = 49).

Variable	Mean	Standard Deviation	95% Confidence Interval	
			Lower	Upper
Age (years)	27.67	7.10	25.66	29.69
M (D)	−0.86	1.62	−1.32	−0.41
J0 (D)	−0.01	0.31	−0.09	0.08
J45 (D)	−0.03	0.15	−0.07	0.02

M: spherical equivalent; J0 and J45: Jackson crossed cylinder lenses, representing the three components of refractive error in power vector notation; D: diopters.

**Table 2:** Data comparison between the pre- and post-exercise intraocular pressure values in the study participants (*n* = 49).

	Pre-Exercise	Post-Exercise	Δ%	*p*-Value	Cohen’s *d*_unb_
IOP (mmHg)	15.28 ± 0.95 [14.78–15.83]	14.30 ± 1.61 [13.93–14.97]	−6.27	<0.001	0.73
CCT (microns)	557.34 ± 35.51 [544.78–566.05]	557.91 ± 35.23 [545.98–566.94]	0.19	0.229	0.03

Post hoc tests’ outcomes with Bonferroni adjustments are presented for intraocular pressure (IOP) and central corneal thickness (CCT). Results are displayed as mean ± standard deviation [95% confidence interval] and percentage of change (Δ%). Cohen’s d represents the effect size of the pre- and post- differences, being *d*_unb_ < 0.50 a small effect, 0.50 ≤ *d*_unb_ ≤ 0.79 a moderate effect, and *d*_unb_ ≥ 0.80 a large effect.

**Table 3:** Data comparison between the pre- and post-exercise intraocular pressure values, according to the sex of the participants (males, *n* = 18; females, *n* = 31).

	Group	Pre-Exercise	Post-Exercise	Δ%	*p*-Value	Cohen’s *d*_unb_
IOP (mmHg)	Male	15.60 ± 1.31 * [15.28–16.04]	13.82 ± 2.29 * [13.06–14.38]	−11.41 **	<0.001	1.02
	Female	14.91 ± 1.04 [15.11–15.71]	14.73 ± 1.81 [14.66–15.70]	−1.20	0.312	0.15
CCT (microns)	Male	546.94 ± 57.95 [526.11–559.51]	546.66 ± 57.13 [526.93–559.85]	0.09	0.395	0.01
	Female	568.02 ± 45.73 [554.84–581.20]	569.53 ± 45.09 [556.54–582.52]	0.27	0.007	0.03

Post hoc tests’ outcomes with Bonferroni adjustments are presented for intraocular pressure (IOP) and central corneal thickness (CCT). Results are displayed as mean ± standard deviation [95% confidence interval] and percentage of change (Δ%). * and ** characterize statistically significant and highly statistically significant differences between sexes, respectively. Cohen’s d represents the effect size of the pre- and post- differences, with *d*_unb_ < 0.50 being a small effect, 0.50 ≤ *d*_unb_ ≤ 0.79 a moderate effect, and *d*_unb_ ≥ 0.80 a large effect.

**Table 4:** Data comparison between the pre- and post-exercise intraocular pressure values, according to the age of the participants (young adults [minor or equal to 25 years], *n* = 21; adults [older than 25 years], *n* = 28).

	Group	Pre-Exercise	Post-Exercise	Δ%	*p*-Value	Cohen’s *d*_unb_
IOP (mmHg)	Young adults	15.27 ± 1.30 [14.89–15.64]	14.88 ± 2.21 * [14.25–15.52]	−2.55	0.154	0.21
	Adults	15.25 ± 1.39 [14.84–15.65]	13.71 ± 2.37 [13.03–14.40]	−10.10	<0.001	0.78
CCT (microns)	Young adults	564.62 ± 58.28 [547.78–581.46]	564.95 ± 57.74 [548.27–581.64]	0.06	0.605	0.00
	Adults	550.05 ± 62.57 [531.97–569.13]	550.87 ± 62.00 [532.95–568.78]	0.15	0.243	0.01

Post hoc tests’ outcomes with Bonferroni adjustments are presented for intraocular pressure (IOP) and central corneal thickness (CCT). Results are displayed as mean ± standard deviation [95% confidence interval] and percentage of change (Δ%). * characterize statistically significant differences between age groups. Cohen’s d represents the effect size of the pre- and post- differences, with *d*_unb_ < 0.50 being a small effect, 0.50 ≤ *d*_unb_ ≤ 0.79 a moderate effect, and *d*_unb_ ≥ 0.80 a large effect.

**Table 5:** Data comparison between the pre- and post- exercise intraocular pressure values, according to the baseline IOP of the participants (lowest [≤14.00 mmHg], *n* = 18; medium [14.01 to 16.99 mmHg], *n* = 17; highest [≥17.00 mmHg], *n* = 14).

	Group	Pre-Exercise	Post-Exercise	Δ%	*p*-Value	Cohen’s *d*_unb_
IOP (mmHg)	Low	13.42 ± 1.43 ** [13.00–13.83]	12.91 ± 2.50 ** [12.19–13.63]	−3.80 ^3^	0.114	0.25
	Medium	15.75 ± 1.44 ** [15.33–16.17]	14.56 ± 2.53 * [13.83–15.29]	−7.56	0.001	0.57
	High	17.44 ± 1.46 [17.02–17.86]	15.88 ± 2.56 [15.14–16.61]	−8.95	<0.001	0.74
CCT (microns)	Low	551.58 ± 63.13 [533.39–569.77]	552.70 ± 62.24 [534.77–570.63]	0.20	0.136	0.02
	Medium	552.03 ± 63.86 [533.63–570.42]	553.24 ± 62.97 [535.10–571.38]	0.22	0.111	0.02
	High	562.65 ± 64.79 [543.98–581.31]	563.44 ± 63.88 [545.03–581.84]	0.14	0.303	0.01

Post hoc tests’ outcomes with Bonferroni adjustments are presented for intraocular pressure (IOP) and central corneal thickness (CCT). Results are displayed as mean ± standard deviation [95% confidence interval] and percentage of change (Δ%). * and ** characterize statistically significant and highly statistically significant differences with the rest of the groups, respectively. ^3^: significant differences with Group 3 (high baseline IOP). Cohen’s d represents the effect size of the pre- and post-differences, with *d*_unb_ < 0.50 being a small effect, 0.50 ≤ *d*_unb_ ≤ 0.79 a moderate effect, and *d*_unb_ ≥ 0.80 a large effect.

**Table 6:** Regression analyses.

Model	Predictor	Unstandardized Coefficients		Standardized Coefficients	*t*	Sig.	Adj. R^2^	△R^2^	Durbin-Watson
		B	S.E.	β					
1	(Constant)	−2.799	1.035		−2.704	0.010	0.358	0.284	1.975
	Age	−0.036	0.026	−0.164	−1.392	0.171			
	Sex	1.800	0.372	0.569	4.835	0.000			
2 *	(Constant)	−0.606	3.059		−0.198	0.844	0.433	0.096	
	Age	−0.035	0.024	−0.158	−1.429	0.160			
	Sex	1.430	0.383	0.452	3.730	0.001			
	Baseline IOP	−0.270	0.096	−0.322	−2.817	0.007			
	Baseline CCT	0.005	0.005	0.104	0.892	0.377			

IOP: Intraocular pressure; CCT: Central corneal thickness; * Retained model; B = Unstandardized effect coefficient; S.E. = Standard Error; β = Standardized effect coefficient (Beta can be interpreted as controlling for the effects of other variables); t = Value of the Student’s *t*-test; Sig = *p*-value of the test; Adj. R^2^ = Adjusted R-square; △R^2^ = Changes in R-square.

## Data Availability

The data presented in this study are available on request from the corresponding author.
